# Polyethyleneimine (PEI) Mediated siRNA Gene Silencing in the *Schistosoma mansoni* Snail Host, *Biomphalaria glabrata*

**DOI:** 10.1371/journal.pntd.0001212

**Published:** 2011-07-12

**Authors:** Matty Knight, Andre Miller, Yijia Liu, Puthupparampil Scaria, Martin Woodle, Wannaporn Ittiprasert

**Affiliations:** 1 Biomedical Research Institute, Rockville, Maryland, United States of America; 2 Aparna Biosciences Corporation, Rockville, Maryland, United States of America; University of Queensland, Australia

## Abstract

An *in vivo*, non-invasive technique for gene silencing by RNA interference (RNAi) in the snail, *Biomphalaria glabrata*, has been developed using cationic polymer polyethyleneimine (PEI) mediated delivery of long double-stranded (ds) and small interfering (si) RNA. Cellular delivery was evaluated and optimized by using a ‘mock’ fluorescent siRNA. Subsequently, we used the method to suppress expression of Cathepsin B (CathB) with either the corresponding siRNA or dsRNA of this transcript. In addition, the knockdown of peroxiredoxin (Prx) at both RNA and protein levels was achieved with the PEI-mediated soaking method. *B. glabrata* is an important snail host for the transmission of the parasitic digenean platyhelminth, *Schistosoma mansoni* that causes schistosomiasis in the neotropics. Progress is being made to realize the genome sequence of the snail and to uncover gene expression profiles and cellular pathways that enable the snail to either prevent or sustain an infection. Using PEI complexes, a convenient soaking method has been developed, enabling functional gene knockdown studies with either dsRNA or siRNA. The protocol developed offers a first whole organism method for host-parasite gene function studies needed to identify key mechanisms required for parasite development in the snail host, which ultimately are needed as points for disrupting this parasite mediated disease.

## Author Summary

Freshwater snails are important in the transmission of schistosomiasis. As part of an integral control effort to combat the spread of schistosomiasis new intervention tools are being sought. One method is to interrupt the transmission of the causative schistosome parasite during the intra-molluscan phase of its development. Gene-silencing technology involving the use of dsRNA have used an injection route to disrupt gene translation in the *Schistosoma mansoni* snail host, *Biomphalaria glabrata* in an effort to investigate how inhibition of various transcripts can affect the dynamics of the snail/parasite interaction. These studies have been helpful in showing us that a gene-silencing pathway that uses dsRNA indeed exists in snails but the injection method previously utilized is impractical, especially when working with juvenile snails. To make the use of gene silencing technology more widely applicable to functional gene studies in snails, we have developed a more convenient soaking method that uses a cationic carrier polyethylene amine (PEI) to deliver dsRNA or siRNA into juvenile snails. Using this method we show the successful knockdown at both RNA and protein levels of the *B. glabrata* peroxiredoxin (Prx) gene. The method was also evaluated for silencing the Cathepsin B (CathB) gene in the snail.

## Introduction

*Biomphalaria glabrata* is an intermediate snail host that transmits the digenean platyhelminth parasite, *Schistosoma mansoni*, in the Western Hemisphere. This snail host is easily maintained in the laboratory outside of its natural environment and, therefore, serves as a useful model organism for conducting studies aimed at unraveling the complex biology that underlies the snail's relationship with the schistosome parasite.

Schistosomiasis, the disease caused by the parasite, is prevalent in several countries of the developing world where it is estimated that at least 200 million people are chronically infected [1], [2]. Significant progress is being made towards the identification of genes that govern the snail/schistosome interaction. Accordingly, several genes that are either up or down regulated in the snail, early after parasite infection have now been identified [3], [4], [5]. Among these are genes involved in innate defense and stress response. A genome project for *B. glabrata* is also near completion [6]. It is hoped that all these advances will lead eventually to the development of novel tools for halting infection at the snail stage of the parasite's life cycle. For this disease transmission blocking strategy to come to fruition, however, we need a better understanding of what genes/cellular pathways in the snail host can be interfered with to bring about subsequent disruption of the parasite's development.

To investigate what gene expression and/or molecular pathways are involved in the snail host/parasite relationship, either enabling or disabling a viable schistosome infection, the technology of RNA interference (RNAi) to specifically silence gene expression in the snail host should help to uncover genes/pathways (in the snail host) that are essential for schistosome development. Fundamentally, it is also possible to envision that this technology might help us to identify conserved molecular pathways that are utilized by the parasite for its survival in both snail and definitive hosts, providing us with an alternative approach towards the identification of new targets for either drug or vaccine development.

All previous studies that have reported successful gene -silencing by RNAi technology in mollusks have been accomplished by an injection approach. For instance, in 2006 by Jiang *et al.* [7] were able to knockdown the expression of the snail defense lectin gene, FREP 2 by directly injecting the corresponding dsRNA of this molecule into the snail hemolymph. Similarly, in another pulmonate gastropod, *Lymnaea stagnalis*, the injection of dsRNA corresponding to the transcript for nitric oxide synthase in this pond snail was shown to cause the reduction in expression of this gene, affecting the feeding behavior of treated snails [8]. Both these studies paved the way in showing that a post-transcriptional gene silencing (PTGS) mechanism, mediated by RNA interference, was indeed operational in freshwater snails, as had been extensively shown in other organisms. Recently, the specific knockdown of the *B. glabrata* ortholog of Macrophage Migration Inhibitory Factor (MIF) was demonstrated at the protein level by injecting the corresponding dsRNA of this molecule into the snail, making this the first time that RNAi technology has been shown to suppress protein function in this snail [9]. In the very few RNAi gene-silencing studies that have been performed in mollusks only one thus far has used siRNA, not dsRNA, for mediating the suppression of specific gene expression. Thus in this recent study, Hannington *et al.* [4] were able to show the knockdown of the protein expression of FREP 3 with a concomitant increase in snail susceptibility, demonstrating the functional role of this gene in snail innate immunity.

Since the discovery was made several years ago of the existence of a dsRNA mediated PTGS pathway in the cell, the knockdown of specific genes, using either their corresponding dsRNA or siRNA, to study gene-function has grown exponentially. In schistosomes, for example, the technique has now been used widely to demonstrate the importance of several key genes whose function enables optimum development of larval and adult worms [10], [11]. Furthermore, key parasite enzymes belonging to this gene-silencing network are being cloned and characterized [12]. Contrary to these significant milestones that have been achieved in the parasite, in the snail host, however, virtually no information exists on how this PTGS pathway operates to regulate gene expression. One exception to this paucity of data is the recent identification and mapping, by fluorescent in situ hybridization (FISH), of the *B. glabrata* homolog of P-element induced wimpy testis, *piwi*, a protein that is involved in siRNA mediated silencing of mobile elements in germ cells [13].

Polyethylenimine (PEI) is a cationic polymer that has been widely used as a carrier for the delivery of DNA, dsRNA and proteins into cells [14], [15], [16]. Because the snail secretes mucus, a complex high molecular weight protein glycoconjugate substance that is used for locomotion, as well as forming a natural protective barrier around the organism [17], it is difficult to deliver nucleic acids in solution directly into mollusks. In order to deliver dsRNA through the negatively charged mucus into the snail, we used PEI as a carrier. Linear (jPEI) and non-linear (branched) PEIs were tested for delivery. The decision to evaluate PEI as a possible carrier for the delivery of dsRNA, or siRNA, into the snail was based on prior experience of using this cationic matrix for separating small oligonucleotides [18]. Furthermore, the synthetic PEI polymer has been shown to be a good non-viral delivery system for studying therapeutic siRNA mediated gene silencing in several animal models. The advantage of using PEI is that the complex that is formed with siRNA is a nanoparticle that is protected from degradation (reviewed by Gunther *et al*) [19]. Initially, the PEI delivery method for the snail was optimized with ‘mock’ fluorescent siRNA. Then the selected method was used to evaluate specific knockdown, in a ‘proof of principle’ experiment, of transcripts encoding *B. glabrata* homologs of Cathepsin B (CathB) [20] and peroxiredoxin (Prx) [21]. Using siRNA and dsRNA corresponding to these genes, in combination with linear (jet) PEI, we were able to show specific knockdown of these transcripts in the snail host.

## Materials and Methods

### Snails

Juvenile *B. glabrata* snails of the NMRI stock (2–3 mm in diameter) were used for the study. The snails were maintained in de-chlorinated tap water at room temperature and fed on Romaine lettuce as previously described [22], [23]. Before either siRNA or dsRNA delivery, snails were kept overnight in sterile H_2_O without feeding.

### RNA isolation and dsRNA preparation

RNA was isolated from the whole snail as previously described [24]. To prepare dsRNA for the Prx (Acc. No. FJ176942) and CathB (Acc.no. EU035711) transcripts, we designed primers corresponding to the transcript sequence that additionally contained sequences of T7 polymerase in *cis*. Forward and reverse primer sequences were as follows: (Prx forward primer; spanned position 162–173) 5′-taatacgactcaccCGTCATTTCTAA-3′, (Prx reverse primer; spanned position 539–550) 5′-taatacgactcaccCTTTGGGGTCAA-3′, (CathB forward primer; spanned position 381–392) 5′-taatacgactcaccATGACTGACAGA-3′ and (CathB reverse primer; spanned position 851–862) 5′-taatacgactcaccTTCACTGCGTGT-3′. In this study dsRNA (Myo dsRNA) for myoglobin (Acc.no. U89283) was also prepared and used as mock dsRNA control. The myoglobin primers utilized for dsRNA preparation were as follows: forward primer (spanned position 1229–1240) 5′-taatacgactcaccGGCAAAAAGAAC-3′ and reverse primer (spanned position 3112–3123) 5′-taatacgactcaccAAGAGCACTTTC-3′. The primers (forward and reverse) were designed to encompass two contiguous exons of the myoglobin transcript [25]. To prepare DNA templates for dsRNA synthesis, cDNA made by using total RNA from the whole snail, as previously described [26] was amplified by PCR for 30 cycles with the following conditions: denaturation at 95°C for 30 seconds, annealing at 55°C for 30 seconds, and extension at 72°C for 1 minute, for 30 cycles. Amplicons of the expected size (397 bp) encompassing positions 161 bp to 558 bp of the BS-90 stock Prx sequence (accession no. FJ176942, [21]) were purified by using either ‘gene clean’ (MPBIO, Solon, OH) or by using a Sephacryl S-200 microspin column (GE Healthcare, UK) according to the manufacturer's instructions. Purified templates were examined qualitatively by agarose gel electrophoresis in TBE buffer as previously described [24] and quantified by a NanoDrop 1000 Spectrophotometer (Thermo Scientific). Templates (cDNA) used for dsRNA synthesis were stored at −20°C in sterile H_2_O until they were required. Amplicons synthesized for Cath B and Myo templates were purified, examined quantitatively and re-suspended as described above.

To prepare dsRNA, we used the purified amplified templates from above at a final concentration 20 ng/µl. After denaturing the template for 5 min at 65°C followed by snap -freezing on ice, the following was added to the template on ice in a final volume of 50 µl, 5× transcription buffer (Promega, WI), 2.5 µM rNTPs, 100 mM DTT, 2 µl (80 units) Rnasin (Promega, WI), and 1 µl (19 units) T7 polymerase. Samples were incubated for 2 hrs at 37°C before adding RNAase free DNAase (4 units, RQI, Promega WI), and incubating for a further hour to remove the DNA template. Reactions were inactivated with 1 µl 0.5 M EDTA (pH 8.0), phenol: isoamyl alcohol/chloroform extracted and precipitated with wheat germ transfer RNA (overnight at −20°C) in 2.5× volumes of Ethanol. The dsRNA was recovered by centrifugation at 10,000×*g* for 15 min at 4°C, and the pellet was washed once in 75% ethanol then air-dried. The dried pellet was re-suspended in sterile de-ionized distilled (dd) H_2_0. The solubilized dsRNA was examined, qualitatively by agarose gel electrophoresis, purified by ‘gene-clean’ (MPBIO, Solon, OH) according to the manufacturer's instructions, quantified by using a Nanodrop spectrophotometer, and stored at −70°C until required.

Two small inhibitory RNAs (siRNAs) for the CathB transcript; CathB1 and CathB2 were designed *Silencer* Select Custom Designed siRNAs (http://www5.appliedbiosystems.com/tools/sirna/). Both the CathB siRNAs utilized were synthesized by Applied Biosystems. CathB1 and CathB2 siRNAs spanned the CathB gene coding DNA position 94–112 (propeptide C1 region) targeting the sequence 5′-GCGATGCAGAGATCTTCTA′3 and position 850–868 (catalytic region) targeting the sequence 5′-GACACGCAGTGAAGATCAT-3′, respectively. In order to reduce off-target effects, the selected target sequences showed no match with any other *B. glabrata* and *S. mansoni* sequences as assessed by *Silencer* Select Custom Designed siRNAs using their ‘BLAST and Filter’ function. In addition, *Silencer* Negative control siRNA#1 was also provided by the manufacturer (Ambion, ABI) and used as negative control. Sequence information for accompanying ‘mock’ siRNAs was not provided by the manufacturer. However, according to the manufacturer's instructions, these ‘mock’ siRNAs do not target any gene product. *Silencer* Negative Control siRNAs are validated for use in human, mouse, and rat cells, and have been functionally tested for producing minimal effects on cell proliferation and viability. Supplied with the ‘Cath B gene specific’ siRNAs, the ‘mock’ siRNA was utilized in all assays to determine the efficiency of the CathB siRNA/PEI transfection, and to rule out non-specific effects of siRNA delivery into the snails. ‘Fluorescence siRNA’ sold as ‘block it’ Alexa 555 (Invitrogen, Life Technologies) was used in this study. According to the manufacturer, the Alexa 555 siRNA sequence is not homologous to any known gene and uptake of this fluorescent siRNA was assessed qualitatively by fluorescent microscopy according to the manufacturer's instructions. Each snail treated with the fluorescent labeled-siRNA was kept in the dark throughout the experimental procedure. Although siRNA and dsRNA are, technically both double-stranded RNA, the current convention is to classify them according to the length of nucleotide sequence, with longer oligonucleotides (>21–23 nucleotides) referred to as dsRNA and shorter ones known as siRNA ‘small interfering RNA’.

### PEI mediated delivery of siRNA and dsRNA

The linear PEI commercially sold as Jet PEI (jPEI molecular weight 22 kDa) was purchased from PolyPlus (France) and branched PEI (bPEI) with average molecular weight of 25 kDa was purchased from Sigma-Aldrich (MO, USA) Both reagents were kept at 4°C until required. For RNA delivery, we combined the dsRNA (120 ng) with PEI (93.8 ng of linear or branched) to obtain the complex at a PEI nitrogen/Nucleic acid phosphate (N/P) ratio of 6 [27]. A residue weight of 43 for PEI and 330 for siRNA was used for calculating the required amounts of PEI and siRNA or dsRNA.

The required amount of siRNA (775 ng) or dsRNA (120 ng) was diluted to 250 µl of sterile ddH_2_O in a microcentrifuge tube. The corresponding amount of PEI was also diluted to 250 µl in sterile ddH_2_O in a separate tube. This PEI solution was added to the tube containing the dsRNA (or siRNA) followed by immediate vortexing for 10 sec. The mixture (PEI plus dsRNA) was allowed to incubate to form nanoparticles at room temperature for 30 min before placing washed snails into the mixture. After placing the snails in the tubes the samples were mixed gently and the caps were closed. Using a hypodermic needle, holes were punched into the caps, and samples were kept at room temperature until the end of the experiment (routinely 72 hrs but also longer for up to 4 days). Snails (6–8 snails for each experiment) were incubated individually in the mixture, and all experimental sets were done in duplicate. Six separate biological replicates using dsRNA/PEI and 5 with siRNA/PEI complexes were performed in total. Because the snails had a tendency to initially crawl out of the PEI/dsRNA mixture, tubes were checked periodically to make sure that snails remained in the solution. Of the two carriers, snails were observed to crawl less frequently out of the linear than branched PEI, consequently we used jPEI for all subsequent specific gene-silencing experiments conducted herein. For each experiment, controls were set up as follows: normal untreated snails, control snails incubated in either dsRNA or siRNA alone, mock Myo dsRNA, mock siRNA (negative control siRNA supplied by the manufacturer), mock siRNA/PEI, and PEI only control. At the end of the experiment, snails were removed from the mixture, washed in sterile H_2_O and either processed immediately for RNA or protein isolation. Snails for RNA isolation were stored either in the RNA isolating reagent and stored at −20°C until required.

### Survival of PEI treated snails

Snails (2 to 3 mm in diameter) incubated as described above in either linear or branched PEI (n = 117) for up to 4 days were monitored daily for mortality compared to normal untreated snails (n = 29).

### Microscopy

To determine the uptake of PEI/fluorescent siRNA nanoparticles by microscopy, the outer shell of each snail was removed and the remaining whole body was rinsed twice in sterile water before viewing under the light microscope (magnification ×10). The same image was then examined for fluorescence (green filter). Images were captured using the advanced SPOT camera imaging software (Diagnostic Instruments Inc. Sterling Heights, MI). Fluorescent signal in tissues was detected qualitatively (as presence or absence of fluorescence) from the snail tissue samples.

### Gene expression analysis

To monitor the expression of Prx or CathB genes after dsRNA/siRNA treatment in individual snails, total RNA was extracted from the whole snail using RNAzol RT (Molecular Research Center, Inc) following the manufacturer's instructions. With this method, all contaminating residual DNA was eliminated as previously described [26]. Quantitative real-time PCR (qPCR) was performed using Brilliant II SYBR green QPCR master mix, in a one-step reaction (Stratagene, Agilent) according to the manufacturer's instructions and run by using the ABI7300 Real Time PCR System (Applied Biosystems). Twenty-five microliters of each qPCR mixture contained 80 ng RNA, 12.5 µl Brilliant SYBR green PCR Master Mix, 200 nM of each gene specific primer and 1 µl of Blocking Reverse transcriptase. Primer concentrations for each assay were determined after optimization. In order to avoid the possibility of falsely amplifying any of the original dsRNA/siRNA that was applied, specific primers (forward and reverse) were designed outside the region used to synthesize the original interfering dsRNA product. For Prx, primers were: Prx-F 5′-ATGGCATCCTCTCTGCAAACCGGG-3′, Prx-R 5′-TTAGAGTTCATCGTTAGATTGC-3′, CathB-F 5′-AGCAACACCATTCCACATC-3-′ and CathB-R 5′-ATAGCCTCCGTTACATCC-3′. To assess the degree of knockdown achieved, primers (F 5′-GTCTCCCACACTGTACCTATC-3′, R 5′-CGGTCTGCATCTCGTTTT -3′) for the housekeeping gene actin; Acc.no CO501282 [28] were used as the endogenous standard for normalization. Additional controls for verifying the specificity of gene knockdown included; 1) dsRNA or siRNA alone, 2) PEI alone and 3) Myo dsRNA 4) buffer alone. Three technical replicates of qPCR were performed with two internal controls to assess both potential genomic DNA contamination (no reverse transcriptase added) and purity of the reagents used (no template added). As indicated above, the knockdown experiment was repeated multiple times (5×) for CathB siRNA, and (6×) for CathB dsRNA and Prx dsRNA (n = 5–6) as independent biological replicates, and by two different investigators. The difference in gene transcript levels were calculated by the ΔΔCt method [29] using the actin housekeeping gene to normalize the quantification of targets. For graphical representation, the ΔΔCt values were normalized to controls and expressed as the percentage difference.

### Protein preparation and analysis using enzyme-linked immunosorbent assay (ELISA from juvenile snail hepatopancreas

Since previous studies have shown that the hepatopancreas is the major location of Prx in *B. glabrata*, soluble protein extracts from individual juvenile snails were prepared from this tissue [21]. Using a sharp scalpel blade, under a dissecting microscope, half of the hepatopancreas was immediately homogenized on ice in sterile PBS (pH 7.5) containing a cocktail of protease and phosphotase inhibitors (Sigma-Aldrich). Soluble protein was isolated from the homogenate as previously described [21]. The remaining dissected tissue (from each snail) was snap frozen in liquid nitrogen and processed for RNA isolation as described above. To determine whether the PEI mediated Prx dsRNA-soaking method would successfully produce concomitant knockdown of corresponding protein in the snail, changes occurring in the amount of Prx between either experimental or control snails were measured quantitatively by ELISA titre using previously described mouse polyclonal antibody prepared against *B. glabrata* recombinant Prx [21]. Since only limited amount of protein material was available from the multiple number of tissue samples processed, we found using ELISA instead of Western Blots [30] to determine the degree of knockdown at the protein level to be more practical, less labor intensive, and also cost-effective. For ELISA assays, 100 µl per well of 10 µg/ml of soluble protein in coating buffer (100 mM bicarbonate/carbonate buffer, pH 9.6) was coated in a MaxiSorpflat-bottom 96 well plate (Nunc) at 37°C overnight, then unbound (excess) proteins were washed out 3 times with washing buffer (0.01% gelatin, 0.05% BSA, 0.05% Tween 20 in 100 mM phosphate buffer saline [PBS], pH. 7.2). The remaining binding sites (that would yield false positive results) were blocked by using 1% BSA in PBS at 37°C for 30 min, then washed (3×) in washing buffer as described above. Subsequently, 10-fold dilution (ELISA titres varied from 100–204,800) of antibody either polyclonal antibody of Prx or CathB (100 µl/well) was added into each well and incubated at 37°C for 1 hr. After washing, each well was incubated with donkey anti-rabbit IgG (H&L) HRP (Promega, MA) for Cath B or goat anti-mouse IgG-HRP (Jackson ImmunoResearch Laboratory, PA) for Prx according to the manufacturer's instructions. The colorimetric enzymatic assay was determined by incubation at RT for 30 min with 3-ethylbenzthiazoline-6-sulfonate by ABTS Microwell Peroxidase Substrate System (Kirkegaard&Perry Laboratories, MD), 100 µl/well. The reaction was stopped with 100 µl of 1% SDS solution, and absorbance at 405 nm was read by using a microplate reader (BioRad Laboratories). All assays were done in triplicate and included the following controls: 1) protein extract incubated with only buffer (no antibody negative control), 2) buffer only incubated with antibody (no antigen negative control), Prx recombinant protein incubated with antibody (positive control). The negative control using the protein extract without antibody was to eliminate the possibility that false positive results might occur due to endogenous peroxidase activity present in the extract that might interfere with the assay. These data are expressed as end point titre, with the titre being defined as the highest dilution that yielded an optical density reading greater than twice the background values. The titres were calculated after subtracting the mean absorbance of triplicate wells lacking antigen protein from the absorbance of triplicate wells containing antigen at each antibody dilution. In cases where snails were treated with CathB siRNA or CathB dsRNA commercial rabbit anti-Cathepsin B polyclonal antibody [CathB Ab] (MyBioSource, CA) and anti-Sm31 polyclonal antibody (anti-*S.mansoni* CathB) were used to determine changes in this protein in treated compared to untreated snails.

## Results and Discussion

### PEI mediates the uptake of fluorescent siRNA into whole *B. glabrata* snails

**Figure 1:**
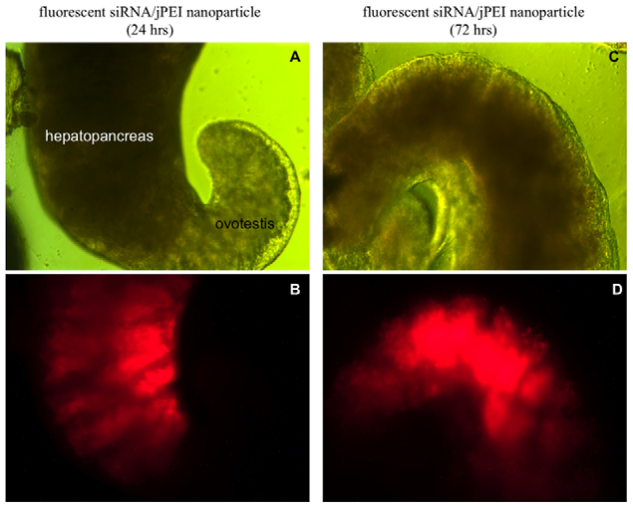
Snail organs transfected with the jPEI/fluorescent siRNA nanoparticles visualized by light and fluorescence microscopy. *Panels A and C*: Images of hepatopancreas and ovotestis regions of juvenile snails that were soaked either in jPEI/Alexa 555 siRNA for either 24 or 72 hrs viewed without fluorescence (10× magnification). *Panels B and D*: Images of the hepatopancreas and ovotestis tissues shown in panels A and C, subjected to fluorescence microscopy as described in Materials and Methods. Note the intense flourescence (red stain) in the hepatopancreas compared to the ovotestis, indicating preferential uptake of the jPEI/fluorecent siRNA nanoparticles into this tissue.

**Figure 2:**
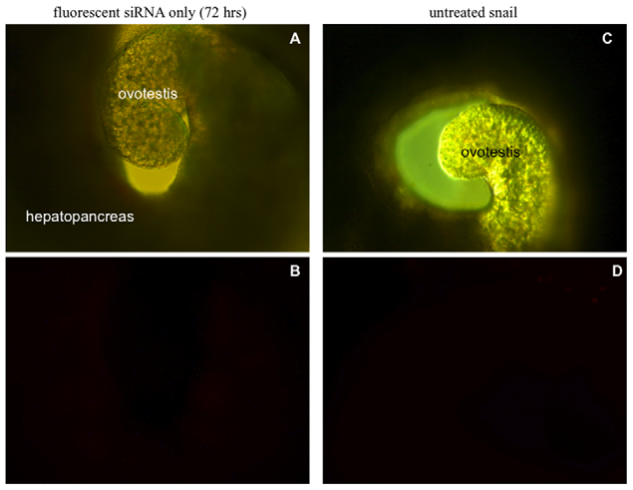
Snail organs transfected with or without the fluorecent siRNA visualized by either light or fluorescence microscopy. *Panels A and C*: Images of snail hepatopancreas and ovotestis tissues of juvenile snails that were either soaked in fluorescent siRNA (A) for 72 hrs or left untreated (C) viewed without fluorescence (10× magnification). *Panels B and D*: The same images of hepatopancreas and ovotestis regions shown in panels A and C, subjected to fluorescence microscopy as described in Materials and Methods. Note the very weak flourescence (red stain) in the hepatopancreas (B) indicating less uptake of the fluorescent siRNA into this tissue occurs without PEI (10× magnification).

To determine whether PEI can mediate the uptake of siRNA into the snail, we tested separately, linear PEI (jPEI) and branched PEI (bPEI) with fluorescent Alexa 555 tagged siRNA, as described in Materials and Methods. Figure 1 shows images of the snail's hepatopancreas and ovotestis regions visualized either under light (A and C), or fluorescent microscopy (B and D) after snails have been incubated for either 24 or 72 hrs at room temperature in fluorescent Alexa siRNA complexed jPEI (Alexa555 siRNA/jPEI nanoparticles). Images of snails incubated in either fluorescent Alexa siRNA alone or untreated normal snails (hepatopancreas and ovotestis regions) also viewed under either light (A and C) or by fluorescent microscopy (B and D) are shown in Figure 2.

In Figure 1, by using fluorescent microscopy (panels B and D), we detected dramatic uptake of fluorescent siRNA/jPEI into the hepatopancreas at 24 and 72 hrs, as shown by the intense fluorescence (red) in this tissue. By comparing the same image, but this time under light microscopy (panels A and C) at 24 and 72 hrs, respectively, it was clear that there was comparatively less fluorescence in the ovotestis. Very little fluorescence was also detected in the head-foot region (data not shown). Results showing low fluorescence in the ovotestis, indicating less delivery of siRNA into this tissue compared to the hepatopancreas were from multiple (×10) experiments and are consistent with other studies. Thus, in linear PEI-mediated delivery of DNA in the mouse, Zou *et al.* [31] detected uptake of the complex essentially in the liver and lungs but not in other organs.

Figure 2 shows images of the hepatopancreas and ovotestis regions of snails that were treated for 72 hrs with the fluorescent siRNA alone (control without PEI) viewed either by light (panel A) or fluorescence microscopy (panel B). Results showed that in the absence of the PEI carrier, only weak fluorescence was detected in the hepatopancreas of this snail. Images in panels C and D of Figure 2 correspond to the hepatopancreas and ovotestis regions of the untreated snail viewed either by light (panel C) or fluorescent (panel D) microscopy. The absence of fluorescence in the control snail in addition to the weak fluorescence detected in snails incubated only with the naked fluorescent siRNA, without PEI (Fig. 2, panel B), supports the conclusion that this carrier mediates the uptake of fluorescence siRNA into the body of the snail by soaking. We have as yet no explanation for how this uptake penetrates the snail's mucus barrier. Additionally, we cannot rule out the possibility that some of the PEI/siRNA nanoparticles might be ingested/swallowed by the snail. Similar intense fluorescence accumulation in the hepatopancreas relative to other tissues was observed by using bPEI instead of jPEI for the delivery (data not shown).

### Cathepsin B siRNA and dsRNA-PEI mediated knockdown of the corresponding snail transcript

**Figure 3:**
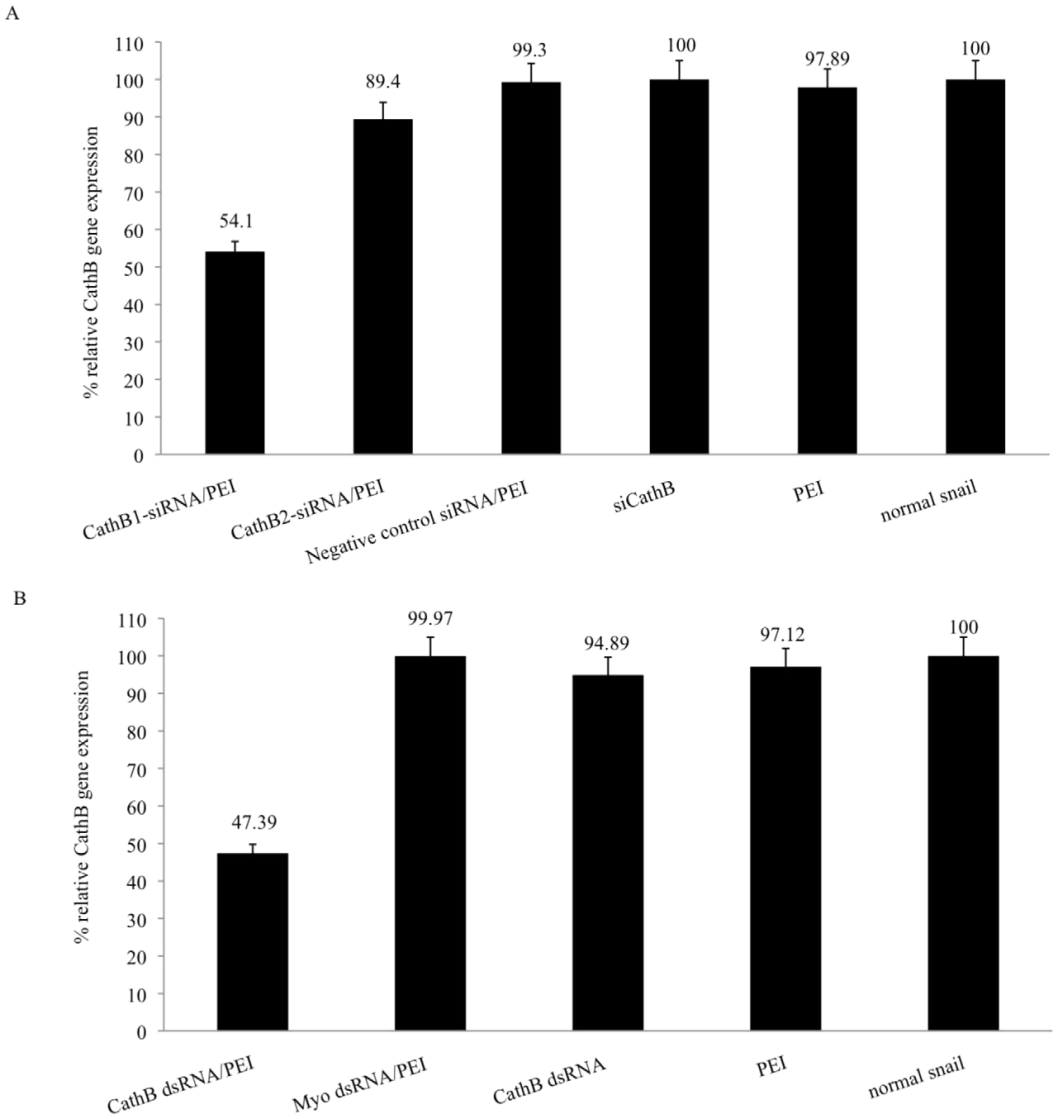
Analysis of Cath B gene expression by real time qPCR. (A) Relative expression of Cathepsin B gene in CathB1 siRNA/jPEI, CathB2siRNA/jet PEI, ‘mock’ CathB siRNA, CathB1siRNA, and PEI treated snails *versus* untreated snails. Treatments were done at room temperature for 72 hrs. (B) Relative expression of Cathepsin B gene in CathB dsRNA/PEI, Myo dsRNA/PEI, CathB dsRNA, and PEI treated *versus* untreated juvenile snails. Treatments were done at room temperature for 72 hrs.

To determine if the delivery protocol developed with fluorescent siRNA (Alexa 555)/PEI nanoparticles can be utilized to target the specific knockdown of a known snail gene we obtained synthetic siRNA designed from different regions of the CathB transcript. We chose siRNA corresponding to conserved regions denoting the propeptide C1 region (siCathB1) and known consensus active site (siCathB2) of this enzyme. PEI complexes of different Cath B siRNA or Cath B dsRNA were tested in a ‘proof of principle’ study, for knockdown of the corresponding CathB transcript. Results showed a knockdown of the CathB transcript; 45.9% and 55.61% suppression (statistically significant differences between the normal snail and other control groups were shown by One-Way ANOVA analysis [*P* value<0.01]) in snails soaked in the CathB1 siRNA/PEI (Fig. 3a) and CathB dsRNA/PEI (Fig.3b) complexes, respectively. In snails soaked in the siCathB2/PEI complex, however, knockdown was found to be less than when the siCathB1/PEI complex was delivered into the snail. This result was unexpected, especially since the commercially purchased siCathB2 encompasses the catalytic region of the enzyme. Other siRNAs designed specifically from the active site of the snail enzyme sequence will be tested in future studies to resolve this unexpected result. In the other snails, no knockdown effect was observed in those treated either with siCathB1 alone (without PEI) or those treated only with PEI. Likewise, the CathB transcript in snails soaked in mock siRNA/PEI remained at about the same level (99.3%) as the level detected in the normal snail (100%). Similarly, as shown in Figure 3b, control snails soaked in naked CathB dsRNA, Myo dsRNA/PEI complexes, and the PEI carrier alone produced no significant knockdown of the CathB transcript.

### Soaking Prx dsRNA with PEI carrier knocks down the corresponding snail transcript and protein

**Figure 4:**
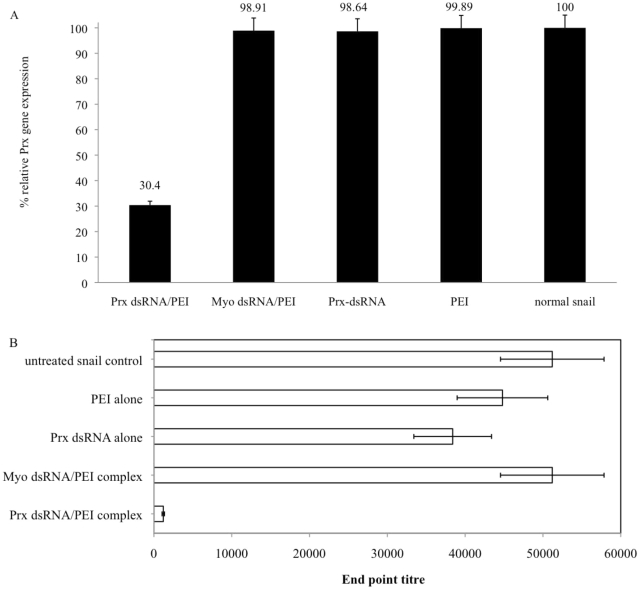
Analysis of Prx expression by real time qPCR and ELISA. (A) Relative expression of peroxiredoxin gene in Prx dsRNA/PEI, Myo dsRNA/PEI, Prx dsRNA alone and PEI treated snails *versus* untreated snails. Treatments were done at room temperature for 72 hrs. (B) Prx protein suppression analyzed by ELISA as described in Materials and Methods with recombinant *B. glabrata* Prx antibody. Each bar represents the geometric mean end point titre ± standard error (SE) of each experimental group as indicated on the y-axis.

To further evaluate the usefulness of the PEI-complex whole organism soaking method, studies were performed with dsRNA corresponding to another previously characterized snail gene, Prx [21]. As shown in Figure 4a, soaking the snails in the *B. glabrata* Prx-dsRNA/PEI complex produced a significant knockdown (70% suppression) of the Prx transcript. In contrast, there was no reduction of the transcript in snails that were soaked in mock Myo dsRNA/PEI, Prx-dsRNA, and PEI alone. The level of transcript in these samples remained almost unchanged, and was comparable to the basal level of the Prx transcript in the normal snail.

To determine whether the above delivery of Prx-dsRNA/PEI complexes would result in the successful suppression of the Prx protein as well, by using ELISA (Fig. 4b), we detected dramatic knockdown of the protein in snails soaked in this complex. The protein suppression observed in Prx dsRNA/PEI treated snails analyzed by one-way ANOVA was statistically significant (*P* value<0.05). Suppression at the protein level was between end-point ELISA titre 800–1,600, compared to either the normal or control groups (end-point ELISA titer between 25,600–51,200). End point titre observed in snails treated with the Prx-dsRNA/PEI complex, reflected a significant reduction in the Prx protein in these snails compared to control snails that were soaked in mock myo-dsRNA/PEI, naked Prx dsRNA, and PEI. Similar ELISA experiments conducted using either commercially available, or *S. mansoni* anti-CathB antibodies showed no cross reaction between the snail CathB homolog and both these reagents. Therefore, investigations to assess the possible knockdown of Cath B transcript, at the protein level, will have to wait for the future generation of specific antibodies for the snail CathB enzyme. In addition, future studies will be done to examine suppression of the enzyme activities of Prx and CathB in order to evaluate whether the demonstrated PEI mediated knockdown of these transcripts translates into disrupting the function of these enzymes as well.

In preliminary studies, the PEI mediated delivery of fluoroscent siRNA into pre-patent snails enabled the detection of fluorescent labeled sporocysts in the infected snails (data not shown). We have as yet, however, no evidence that our PEI delivery protocol will provide a useful avenue for targeting specific sporocyst (parasite) genes for silencing. Initial attempts to use the PEI delivery protocol for siRNA delivery in miracidia have so far been unsuccessful.

### PEI is non-toxic in juvenile *B. glabrata* snails

**Figure 5:**
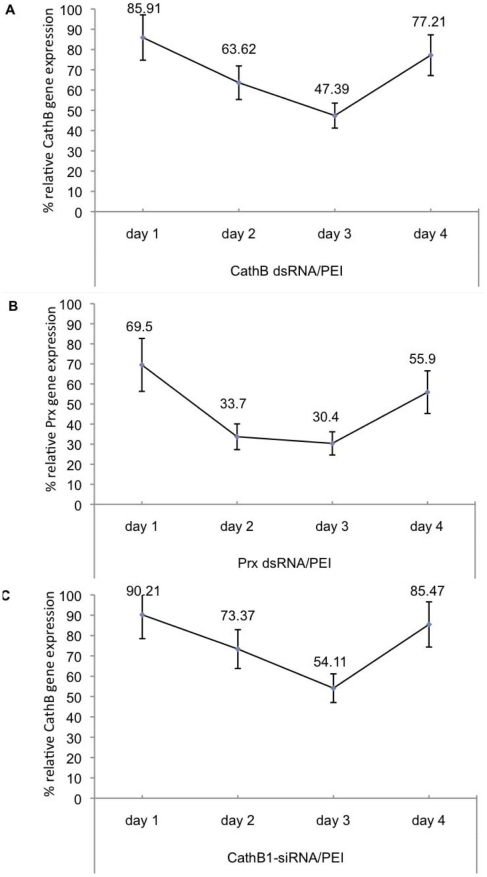
Time course for optimum gene knockdown after soaking snails in either dsRNA/PEI or siRNA/PEI nanoparticles. (A) Relative expression of Cathepsin B gene in juvenile snails soaked for between 1 and 4 days in CathB dsRNA/PEI. (B) Relative expression of peroxiredoxin gene in snails soaked for between 1 and 4 days in Prx dsRNA/PEI. (C) Relative expression of Cathepsin B gene in snails soaked for 1 to 4 days in CathB1-siRNA/PEI. Note in all time course studies that the optimum knockdown of transcripts occurs at day 3 of incubating in either dsRNA/PEI or siRNA/PEI complexes.

**Figure 6:**
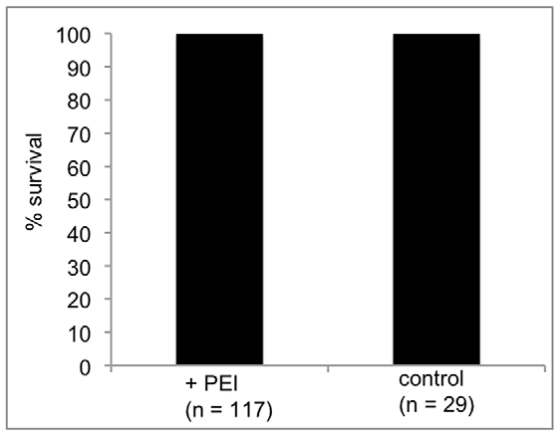
Survival of juvenile snails treated with PEI. Percentage survival of juvenile snails (n = 117) soaked in PEI compared to untreated control snails (n = 29).

In the present study we found that soaking the snails for 1 to 4 days in either dsRNA/PEI or siRNA/PEI resulted in gene suppression within the first 24 hours that extended to 4 days post- treatment (Fig. 5). Thus, in Figures 5a and 5b, we detected significant knockdown of CathB and Prx after 2 and 3 days of soaking in dsRNA/PEI nanoparticles. Similarly, optimum knockdown of CathB occurred following 2 and 3 days of soaking in the siRNACathB/PEI complex (Fig. 5c). These results also showed that after 4 days of soaking, in either dsRNA/PEI or siRNA/PEI, a recovery from the treatment began to occur, indicating, therefore, that the time period for achieving optimum knockdown (using the conditions we employed) occurs 72 hrs after snails have been soaked in the complexes. Furthermore, we detected no mortality under the conditions utilized even though the snails remained in PEI for as long as 4 days. Thus, results in Figure 6 shows the 100% survival of snails (n = 117) treated with PEI *versus* untreated snails (n = 29), indicating that the cationic polymer (both linear and branched) was non-toxic at the concentration utilized in the protocol.

Comparable uptake of siRNA/PEI into adult snails was not examined as part of this work because our current research is primarily focused on elucidating the molecular basis of the more vulnerable juvenile snail host's relationship with the parasite. Since our previous attempts to introduce dsRNA into juvenile snails *via* an injection route produced either inconsistent results or killed the snails, the pressing need for us, therefore, was to develop a reliable and safe gene-silencing tool specifically for snails at this young age and not for adult snails.

By complexing either siRNA or dsRNA to the cationic PEI polymers we have shown in the present study that it is possible to obtain significant knockdown of specific snail transcripts, at both RNA and protein levels, by whole organism soaking. As stated above, most previous studies using RNAi technology in snails have used an injecting route for delivery [7], [8]. The injection method can, however, be cumbersome for an inexperienced investigator, making a straightforward dsRNA delivery method for snails (especially juveniles) until now very challenging and impractical. For this reason, while functional gene analysis with RNAi technology is now used routinely in several organisms, including schistosomes [11] very few molecular functional studies using RNAi have been reported for molluscs. With several genome projects for mollusks now underway, including the *B. glabrata* genome project [6] the need for an easier and more convenient dsRNA delivery approach for future functional genomic studies in these organisms is overdue. The simplicity of the method described here, paves the way as a first step towards overcoming the challenges faced in using RNAi technology routinely in mollusks.

The PEI soaking method was optimized and evaluated using two snail transcripts (Cathepsin B and peroxiredoxin) that we have previously shown to be expressed, preferentially in the snail's hepatopancreas. Both genes are also early and more significantly induced in the resistant *B. glabrata* snail stock, BS-90, compared to the susceptible NMRI snail, in response to *S. mansoni* exposure [20], [21]. With the PEI mediated siRNA and dsRNA gene silencing method developed, we now have the ability to systematically determine the effect of down regulating CathB and Prx as well as other transcripts in the dynamic interplay of the snail- schistosome interaction. Our results showed that the majority of the fluorescent siRNA/PEI complex was detected in the hepatopancreas. It is possible, therefore, that we might have been able to knock- down the two transcripts we chose easily because they are also expressed preferentially in this tissue. In any case, even if limited to this tissue the ability to use a simple and effective whole organism soaking method to conveniently knockdown specific genes in *B. glabrata* by RNAi technology is an important first step in using this proven gene-silencing tool for snail molecular research.
